# Hermansky-Pudlak syndrome type 2: A rare cause of severe periodontitis in adolescents—A case study

**DOI:** 10.3389/fped.2022.914243

**Published:** 2022-07-19

**Authors:** Jun Chen, Yifan Yang, Binjie Liu, Xiaoli Xie, Wenjie Li

**Affiliations:** ^1^Hunan Key Laboratory of Oral Health Research, Hunan 3D Printing Engineering Research Center of Oral Care, Hunan Clinical Research Center of Oral Major Diseases and Oral Health, Xiangya School of Stomatology, Central South University, Changsha, China; ^2^Department of Periodontics, Xiangya Stomatological Hospital, Central South University, Changsha, China; ^3^Deparment of Orthodontics, Xiangya Stomatological Hospital, Central South University, Changsha, China; ^4^Department of Oral Health Science, School of Dentistry, University of Washington, Seattle, WA, United States

**Keywords:** periodontitis, juvenile, Hermansky-Pudlak syndrome type 2 (HPS-2), genetics, adaptor protein complex 3 (*AP3B1*)

## Abstract

**Background and aims:**

Hermansky-Pudlak syndrome (HPS) is an autosomal recessive disorder characterized by oculocutaneous albinism (OCA) and platelet storage pool deficiency. The HPS-2 subtype is distinguished by neutropenia, and little is known about its periodontal phenotype in adolescents. *AP3B1* is the causative gene for HPS-2. A 13-year-old Chinese girl presented to our department suffering from gingival bleeding and tooth mobility. Her dental history was otherwise unremarkable. Suspecting some systemic diseases as the underlying cause, the patient was referred for medical consultation, a series of blood tests, and genetic tests. In this case study, periodontal status and mutation screening of one HPS-2 case are presented.

**Methods:**

Blood analysis including a complete blood count (CBC) and glycated hemoglobin levels were measured. Platelet transmission electron microscopy (PTEM) was performed to observe the dense granules in platelets. Whole-exome sequencing (WES) and Sanger sequencing were performed to confirm the pathogenic variants.

**Results:**

A medical diagnosis of HPS-2 was assigned to the patient. Following the medical diagnosis, a periodontal diagnosis of “periodontitis as a manifestation of systemic disease” was assigned to the patient. We identified novel compound heterozygous variants in *AP3B1* (NM_003664.4: exon7: c.763C>T: p.Q255^*^) and (NM_003664.4: exon1: c.53_56dup: p.E19Dfs^*^21) in this Chinese pedigree with HPS-2.

**Conclusion:**

This case study indicates the importance of periodontitis as a possible indicator of underlying systemic disease. Systemic disease screening is needed when a young patient presents with unusual, severe periodontitis, as the oral condition may be the first of a systemic abnormality. Our work also expands the spectrum of *AP3B1* mutations and further provides additional genetic testing information for other HPS-2 patients.

## Introduction

Hermansky-Pudlak syndrome (HPS, OMIM 203300) is an autosomal recessive disorder, which is characterized by oculocutaneous albinism (OCA), a bleeding diathesis, and, in some individuals, pulmonary fibrosis, colitis, neutropenia, and immunodeficiency (1). There are 11 known subtypes of HPS (2–4). The incidence of HPS type 2 (HPS-2) is <1/1,000,000 (5). To date, ~37 individuals with HPS-2 have been reported in the literature (2, 6, 7). They are of different races, with 2 cases reported from Asia (6, 7). Neutropenia is a characteristic clinical manifestation of HPS-2 (5, 8).

Hermansky-Pudlak syndrome type 2 is associated with biallelic variants in *AP3B1* on chromosome 5q14.1. *AP3B1* gene mutation leads to incomplete formation of adaptor protein 3 (AP-3) complex, resulting in lysosomal-related cell dysfunctions (9, 10): (1) In melanosomes, impaired tyrosinase transport reduces melanin synthesis; (2) The decrease or absence of dense particles in platelets leads to platelet storage pool deficiency, resulting in impaired platelet function (11, 12); (3) In neutrophils, abnormal transport of azurophilic granule leads to impaired cell maturation, resulting in neutropenia (9, 13); (4) Natural killer (NK) cell function is impaired (5, 10).

The diagnosis of HPS-2 is confirmed by pathogenic variants in *AP3B1*, absence of platelet dense granules, and clinical symptoms, including OCA and neutropenia (11, 12).

Periodontitis is one of the prominent manifestations of some systemic diseases and is affected by systemic factors. Some systemic diseases can affect the resistance to bacteria, thus greatly increasing the susceptibility to periodontitis (14). Due to neutropenia and impaired platelet function, many HPS-2 patients, especially young children have gingival bleeding (11, 15–18). However, there are few reports of HPS-2 with severe periodontitis as the prominent manifestation.

In this paper, we reported a case of a 13-year-old Chinese girl who presented to the Department of Periodontics with gingival bleeding and tooth mobility. She was diagnosed with HPS-2 by blood tests, platelet transmission electron microscopy (PTEM), whole-exome sequencing (WES), and Sanger sequencing. Following the medical diagnosis, a periodontal diagnosis of “periodontitis as a manifestation of systemic disease” was assigned to the patient (19). Novel compound heterozygous variants in *AP3B1* (NM_003664.4: exon7: c.763C>T: p.Q255^*^) and (NM_003664.4: exon1: c.53_56dup: p.E19Dfs^*^21) were identified in the proband, inherited from her father and mother, respectively. To the best of our knowledge, these variants have not been reported in previous studies.

## Materials and methods

### Study approval

The protocol conformed to the declaration of Helsinki principles. The study methodologies were approved by the Ethics Committee of Xiangya Stomatological Hospital, Central South University. The proband (the patient) and her parents agreed to the protocol approved by the Institutional Review Board and signed it (Ethic Approval Number: 20210086).

The periodontal examination protocol contained the simplified oral hygiene index (OHI-S), including debris index (DI) and calculus index (CI) (20); periodontal probing examination, including bleeding index (BI) (21), bleeding on probing (BOP), periodontal probing depth (PPD); and imaging examination.

### Peripheral blood analysis

The proband's peripheral blood was collected for complete blood count (CBC) and glycated hemoglobin levels on 12 and 24 July 2021.

### Platelet transmission electron microscopy analysis

Blood samples of the proband were centrifuged in Ethylenediaminetetraacetic acid disodium salt (EDTA-Na_2_) solution, platelets were separated, fixed in 2.5% glutaraldehyde, fixed with conventional osmium acid, dehydrated with ethanol, replaced with acetone, embedded in epoxy resin, and sliced with an ultra-thin cutting machine, and finally observed by PTEM (HT7700; Hitachi, Japan).

### Whole-exome sequencing

Peripheral blood samples of the proband and her parents were collected using the DNesy Blood & Tissue Kit (Qiagen, Valencia, CA, USA) for DNA extraction. Exome capture, high-throughput sequencing, and common variant filtering were provided by Berry Genomics Co., Ltd. (Beijing, China). By using the cBot Cluster Generation System and HiSeq PE Cluster Kit (Illumina, San Diego, CA, USA), the index-coded samples were clustered. After clustering, DNA libraries were sequenced on the Illumina HiSeq 2500 platform (Illumina, San Diego, CA, USA), and 150 bp paired-end reads were generated. By using the 1,000 Genomes Project database (https://www.genome.gov/27528684/1000-genomes-project/), the Chinese Millionome Database (https://db.cngb.org/cmdb/), the Genome Aggregation database (http://gnomad.broadinstitule.org), and the Exome Aggregation Consortium database (http://exac.broadinstitute.org/), the common variants were filtered (frequency ≥ 0.05). After that, unique single-nucleotide polymorphisms (SNPs) were identified. Potential causative variants were screened by the list of genes related to HPS and then predicted by using bioinformatic programs including MutationTaster (http://www.mutationtaster.org/), Polyphen-2 (http://genetics.bwh.harvard.edu/pph2/), and SIFT (http://provean.jcvi.org/index.php). By using Online Mendelian Inheritance in Man (OMIM) (https://www.omim.org), the analyses of gene function, inheritance pattern, and clinical phenotype were conducted. The sequence data has been submitted to the GenBank databases under BioProject ID: PRJNA825281.

### Co-segregation analysis

Primer pairs (*AP3B1* c.763C>T f: AACAAGAGACAAATGAGTCTTCC; *AP3B1* c.763C>T r: GAAGTATGCCCGGACAGAATAG; *AP3B1* c.53_56dup f: CTGCTAAAGAGGCCGAAGG; *AP3B1* c.53_56dup r: ACTGCCAGGTCGGCTCAG) were designed for co-segregation analysis by Integrated DNA Technologies (https://sg.idtdna.com/pages). The target fragments were amplified by polymerase chain reaction (PCR) and analyzed by the ABI 3100 Genetic Analyzer (ABI, Foster City, CA, USA).

## Results

### Clinical manifestation

The proband, female, 13 years old, presented to the Department of Periodontics, Xiangya Stomatological Hospital of Central South University on 12 July 2021 with a chief complaint of gingival bleeding, tooth mobility for almost 3 years. She brushed her teeth twice a day.

Past medical history showed signs of OCA, neutropenia, and congenital pulmonary edema. The proband has a visual acuity of 20/200, photophobia, nystagmus, wandering eye movements, and a lack of visual attention. Eye surgery has been done. There was no history of the following: hypertension, diabetes, etc., infectious diseases such as tuberculosis, anticoagulant drugs, drug allergy, smoking or drinking, bruxism, and prosthodontic or orthodontic treatment. The proband had a natural birth at term. Her parents are not in consanguineous marriage and are in good health.

**Figure 1:**
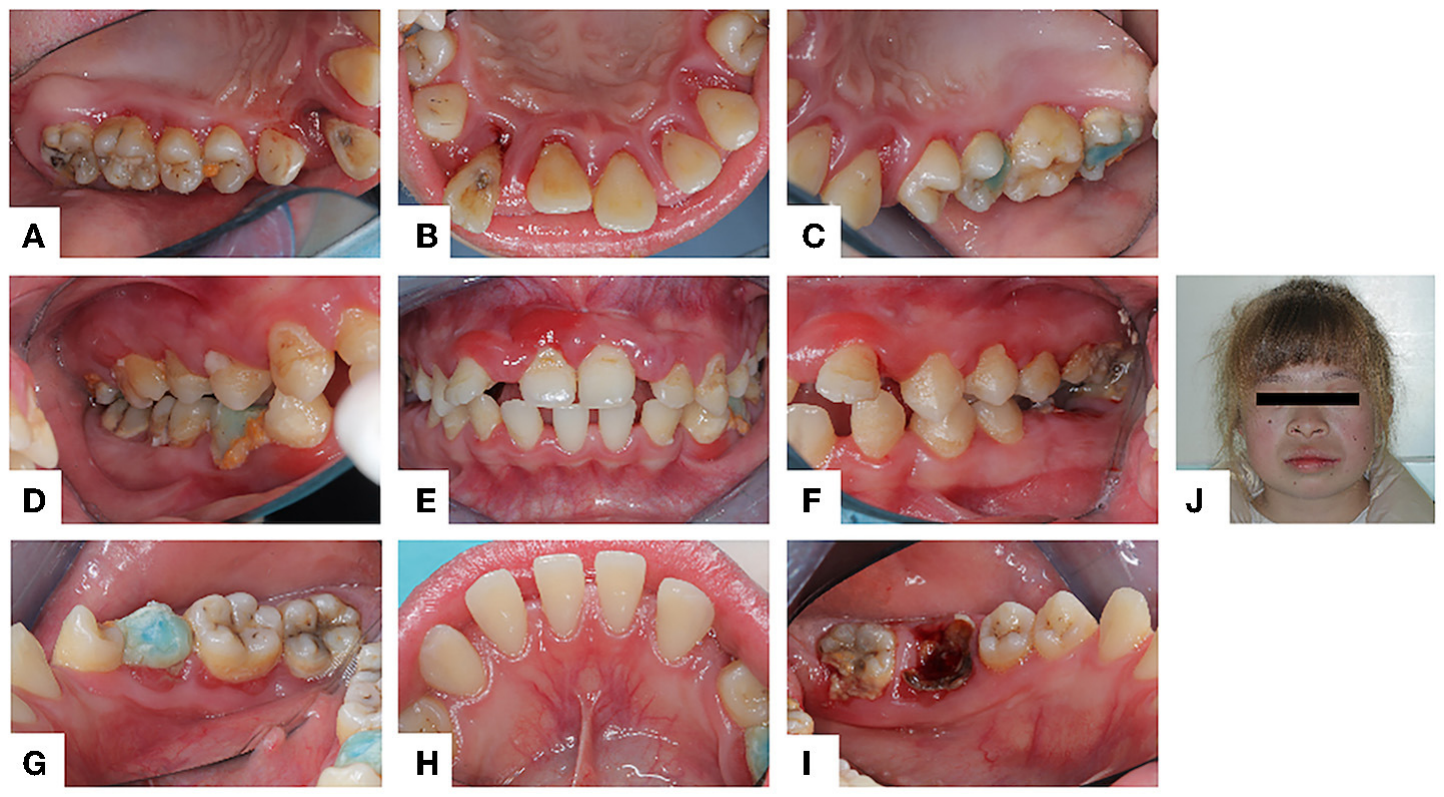
Intraoral and facial photo (July 12, 2021): **(A–I)** The gums were extensively red and swollen. Abduction and displacement of maxillary anterior teeth. **(J)** The proband has blonde hair and whole-body skin whiteness.

**Figure 2:**
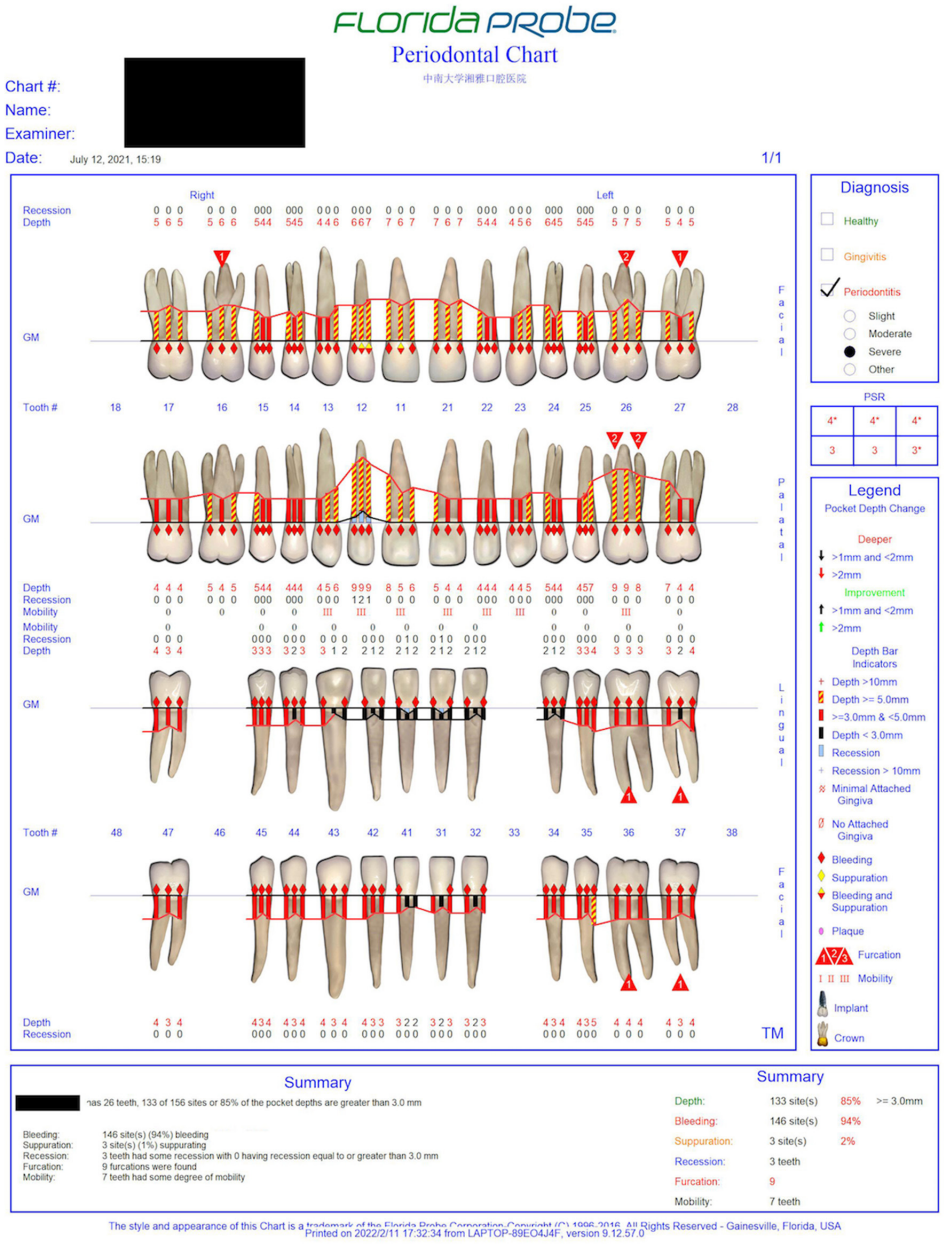
The proband's periodontal chart at the initial consultation.

The oral examination showed normal labial and buccal mucosa, normally hard and soft palates, and oropharynx. The oral hygiene was not good. The gums were red and swollen (Figure 1). The proband's periodontal status was examined using Florida probe® (Florida Probe Corp, Gainesville, FL). The DI reached 0–2, the CI reached 0–2, and BI reached 2–4. The percentage of positive BOP sites was 94%. The PPD was 1–9 mm, and PPD ≥ 3 mm accounted for 85%, among which PPD ≥ 5 mm accounted for 33%. The clinical attachment level (CAL) was 0~7 mm. The mobility of teeth #11, #12, #13, #21, #22, #23, #26 reached degree III. Tooth #33 was congenital missing. Details can be found in the periodontal chart(Figure 2).

General physical examination showed the proband has white skin and blonde hair (Figure 1). She has no intelligence or mental abnormality. She has a normal gait, posture, and motor coordination. Her temporomandibular joints, cervical lymph nodes, and neck were clinically normal.

**Figure 3:**
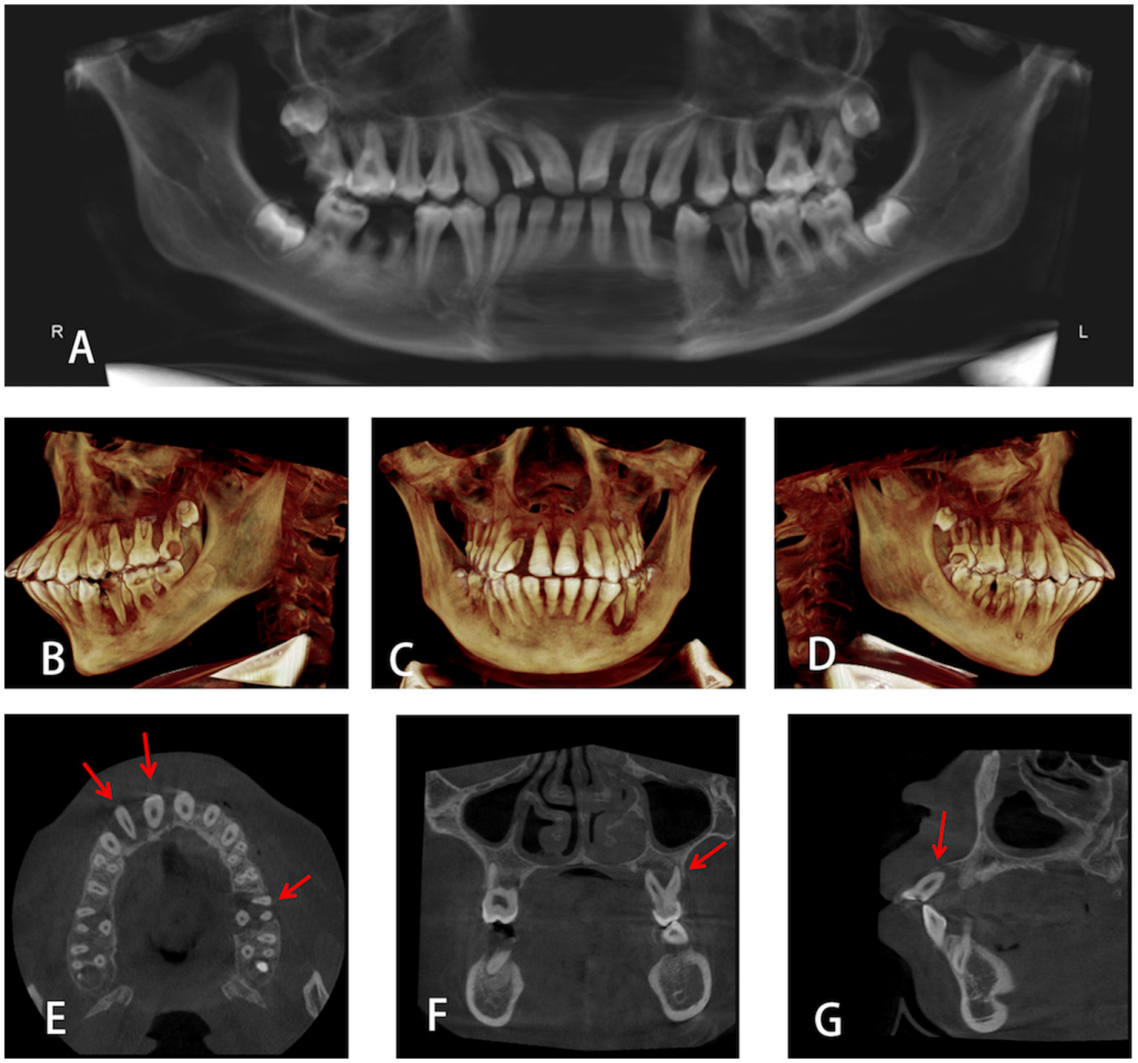
**(A)** Panoramic radiograph of the proband. **(B–D)** 3D Volumetric reconstructive cone-beam computed tomographic (CBCT) images of the whole mouth from different directions. **(E–G)** Alveolar bones of teeth #12, #11, and #26 were absorbed to the root apex.

Imaging examination (Cone-beam computed tomographic, CBCT) showed that most teeth have moderate to severe resorption of alveolar bone, which is inconsistent with age at 13 years. Alveolar bones of teeth #12, #11, and #26 were absorbed to the root apex and there was almost no alveolar bone support in the root (Figure 3).

### Peripheral blood analysis

On July 12, blood analysis showed that the white blood cell count (WBC) (10^∧^9/L) was 1.30, absolute neutrophil count (ANC) (10^∧^9/L) was 0.17, and neutrophil ratio (NEUT) (%) was 13.12. On July 24, a second blood test showed that the WBC (10^∧^9/L) was 1.77, ANC (10^∧^9/L) was 0.55, and NEUT (%) was 31.12. Glycated hemoglobin levels were normal. Upon further examination and blood analysis by the pediatric hematologist, it was noted that the patient's past CBC tests indicated that her white blood cell and neutrophil count were consistently low. Since the neutrophil count remained low without a periodic change, a diagnosis of cyclic neutropenia was quickly ruled out.

### Platelet transmission electron microscopy analysis

**Figure 4:**
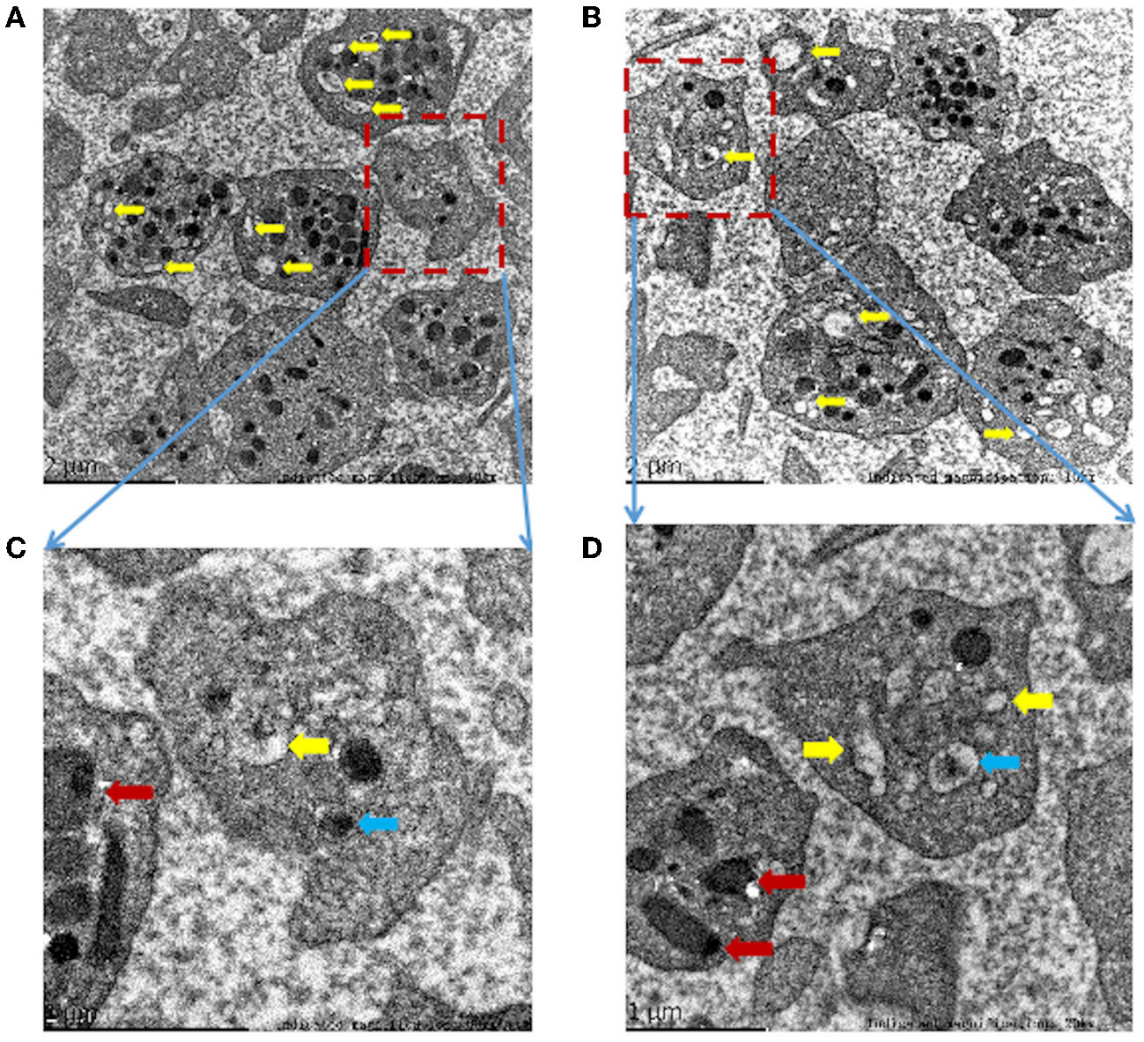
**(A–D)** Morphological analysis of platelets by platelet transmission electron microscopy (PTEM) shows multiple empty sacks (yellow arrows) in the proband's platelets without dense granules. Occasionally, abnormally dense granules with irregular shapes, decreased the content density and increased clear space (blue arrow). The platelet lysosomes (red arrow) increase in volume. Indicated magnification: **(A)** 10kx, **(B)** 10kx, **(C)** 30kx, **(D)** 20kx.

Dense granules are the smallest granules in platelets, which contain strongly electron-dense cores and transparent spaces surrounded by single membranes (22). A total of 4–8 dense granules in each platelet can be seen in healthy people under the PTEM (12, 22). However, the morphological analysis of the proband's platelets under the PTEM showed that multiple vacuoles were seen in the platelets, and dense granules were significantly reduced or even absent. The clear space in the remaining dense granules was enlarged and irregular in shape (Figures 4A–D). The volume of lysosomes in platelets increased (Figures 4C,D).

### Genetic analysis

Whole-exome sequencing (WES) yielded 10.5 Gb of data with 96.96% coverage of the target region and 95.41% of the target covered at least 10 ×. After a series of database analyses and filtering, a set of eight heterozygous variants in six genes in the proband were identified (Table 1). By analyzing the bioinformatic prediction, inheritance pattern, OMIM clinical phenotypes, and American College of Medical Genetics (ACMG) classification (23) of these six genes, the *AP3B1* variant (c.763C>T: p.Q255^*^) and *AP3B1* variant (c.53_56dup: p.E19Dfs^*^21) were supposed to be the causative variants in the proband.

**Table 1:** Eight heterozygous variants in six genes identified by whole-exome sequencing (WES) in the proband.

**Gene**	**Variant**	**MutationTaster_pred**	**Polyphen2_HVAR_pred**	**SIFT_pred**	**1000g2015aug_all**	**ExAC_ALL**	**gnomAD_exome_ALL**	**OMIM clinical phenotype**	**American College of Medical Genetics classification (ACMG)**
WDTC1	c.1468+22G>A	–	–	–	0.0113818	0.013	0.0144884	–	
LAMP1	c.112G>C	D	B	D	–	1.653e-05	2.40429e-05	–	pm2
LAMP1	c.562+4G>A	–	–	–	0.00319489	0.0009225	0.000821249	–	pp3
SP6	c.482T>C	D	B	T	–	2.523e-05	1.12408e-05	–	pm2 bp4
AP3D1	c.906+9C>T	–	–	–	0.000599042	0.0002106	0.000183248	617050	pm2
AP3B1	c.763C>T	A	–	–	–	–	–	608233	pvs1 pm1 pm2 pp3
VPS33A	c.1165-50_1165-47del	–	–	–	–	–	–	617303	pm2 pm3_supporting
AP3B1	c.53_56dup	–	–	–	–	–	–	608233	pvs1 pm2

*MutationTaster_pred: A: disease_causing_automatic; D: disease_causing; N: polymorphism; P: polymorphism_automatic.*

*Polyphen2_HVAR_pred: D: Probably damaging (≥0.957), P: Possibly damaging (0.453 ≤ pp2_hdiv ≤ 0.956); B: Benign(pp2_hdiv ≤ 0.452).*

*SIFT_pred: D: Deleterious (sift ≤ 0.05); T: tolerated (sift > 0.05).*

*1000g2015aug_all: Allele frequency of the mutated base at this mutation locus in all populations of the 1000 Genomes Project.*

*ExAC_ALL: ExAC_ALL Database allele frequency of the mutated base at this variant locus in all populations.*

*gnomAD_exome_ALL: Allele frequencies of this variant in all populations of the gnomAD exon database.*

*phenotype_omim: This mutation corresponds to the OMIM number phenotype in the OMIM database.*

*ACMG: pathogenic: PVS1>PS1>…>PS4>PM1-6>PP1-5; benign: BA1>BS1-4>BP1-7. PVS, pathogenic very strong; PS, pathogenic strong; PM, pathogenic moderate; PP, pathogenic supporting; BA, benign strong; BP, benign supporting*.

**Figure 5:**
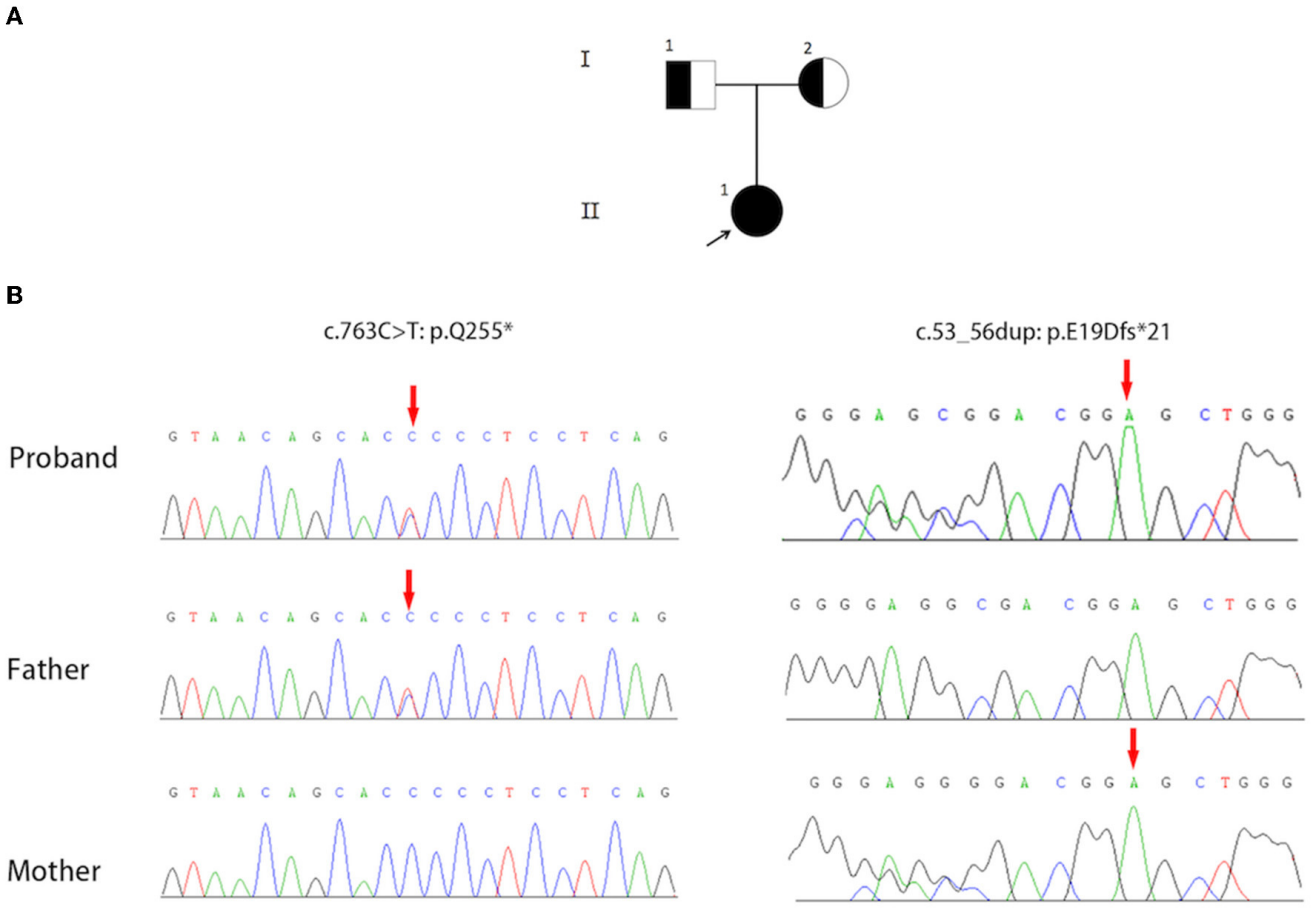
**(A)** The pedigree contains three family members and two generations of Hermansky-Pudlak syndrome type 2 (HPS-2) (I1, I2, II1). The square represents the male, and the circle represents the female. The filled circle (arrow) represents the proband. A half-filled square or circle represents the carrier. **(B)** Sequence Chromatograms show the compound heterozygous mutations (c.763C>T: p.Q255*) and (c.53_56dup: p.E19Dfs*21) in the *AP3B1* gene.

Sanger sequencing results confirmed that the proband carried compound heterozygous variants in *AP3B1* (NM_003664.4: exon7: c.763C>T: p.Q255^*^) and (NM_003664.4: exon1: c.53_56dup: p.E19Dfs^*^21), and was inherited from her father and mother, respectively (Figure 5). So far, a total of 37 HPS-2 patients have been reported (2, 6, 7), and 31 pathogenic mutations have been reported (Table 2). The two alleles (c.763C>T: p.Q255^*^) and (c.53_56dup: p.E19Dfs^*^21) in HPS-2 have not been reported previously.

**Table 2:** Reported *AP3B1* pathogenic gene variants causing Hermansky-Pudlak syndrome type 2 (HPS-2).

**mRNA NM_003664.4**	**Amino acid NP_003655.3**	**Gender/age**	**Oral presentation**	**References**
c.2T > G	p.Met1Arg	M/90m	NA	(24)
c.60 delG (*)	p.L20fsX41	NA/1.25y, 2y	NA	(25)
c.155_158delAGAG	p.Glu52Alafs*11	M/NA	NA	(26)
c.177delA	p.Lys59Asnfs*5	M/52y	NA	(27)
c.205T > C	p.Leu102Pro	NA/11y	NA	(25)
c.716G > A	p.Trp239*	F/1m	NA	(27)
c.904A > T	p.Arg302*	M/2y	Recurrent oral thrush	(28)
c.1063_1064delCAinsSTATCAATATC^g^	p.Gln355Tyrfs*6	Pt1: F/7y, Pt2:M/4y^b^	NA	(10)
c.1095+5G > A	IVS10+5G>A	M/3y	Gingivitis, gum bleeding, excessive bleeding with dental procedures	(16)
c.1168–1G > C^c^	IVS11–1G>C	M/27y, M/22y	NA	(17, 29)
c.1473+6T > C	IVS14+6T>C	NA	NA	(9)
c.1525C > T^e^	p.Arg509*	M/14y	Gingivitis	(30)
c.1619dupG	p.Ala541Serfs*25	NA	NA	(9)
g.del8168-bp	del exon 15	M/15y, M/21y	Pt2: aggressive periodontitis^#^	(15)
g.del1872-bp	del exon 15	F/14y	NA	(31)
del exon 16	–	NA/16y	NA	(25)
c.1739T > G*^d^	p.Leu580Arg	M/27y, M/22y	NA	(29)
c.1754delT	p.Val585Glufs*6	F/4m	NA	(27)
c.1789dupA^h^	p.Ile597Asnfs*12	Pt1: F/7y, Pt2:M/4y	NA	(10)
c.1839_1842delTAGA	p.Asp613Glufs*38	F/4m^a^	NA	(15, 27, 31)
c.1975G > T^f^	p.Glu659*	M/11y	Gingivitis	(30)
c.2041G > T	p.Glu681*	NA/2y	NA	(25, 32)
g.del624-bp c.del2077_2164	p.Glu693Valfs*13	F/NA	NA	(26)
c.2546T > G	p.Leu849*	M/19y	NA	(31)
c.2702C > G	p.Ser901Cys	M/12m	NA	(27)
c.2770delC(*)	p.Leu924Pefs*3	NA/2.5y, 11y	NA	(25)
c.2944delC	p.Leu982Cysfs*19	F/15y	NA	(31)
c.3222_3223delTG(*)	p.Lys1076Asnfs*60	NA/5y, 13y	NA	(25, 31, 33)
Inv(5)p15.1-q14.1	–	F/70m	NA	(34)
c.188T > A c.2546 > A	p.M63K p.L849X	F/1y	NA	(6)
C.1363+1G > A	–	M/6m	NA	(7)

*–, unreported; Pt, Patient.*

*^a^This patient had c.1839_1842delTAGA mutation in addition to c.177delA.*

*^b^These two patients had c.1063_1064delCAinsSTATCAATATC mutation in addition to c.1789dupA.*

*^c/d^They are the same patient. They showed compound heterozygous mutations in AP3B1 (c.1166-1228del [p.390-410del] and c.1739T>G [p.L580R]). The mutation was originally described as del63-bp, but later research on it found that is c.1168-1G>C (IVS11-1G>C) leading to aberrant splicing (17).*

*^e/f^They are the same patient.*

*^g/h^They are the same patient.*

*This mutation involves two patients with similar clinical manifestations and is described together.

*^#^This patient wore orthodontic brackets, and this study did not rule out possible damage to periodontal tissue caused by orthodontic factors*.

## Discussion

In this paper, we reported the periodontal phenotype of a 13-year-old patient with HPS-2 who has a novel complex heterozygous *AP3B1* mutation. The patient manifests with severe periodontitis. The unusual occurrence of severe periodontitis in children and adolescents invariably calls for a thorough investigation of the patient's overall medical condition. Periodontitis may be an important warning sign of systemic illness (35).

Mutations in *AP3B1* lead to incomplete formation of the AP-3 complex, resulting in lysosomal-related cell dysfunctions, including neutropenia, impaired platelet function, and impaired NK cell function (12, 15). Neutropenia is characterized by a decrease in the absolute number of circulating neutrophils and increased susceptibility to infections, including periodontitis (27).

Periodontitis is an inflammatory reaction caused by subgingival biofilm. Subgingival biofilm contains a variety of microbial communities that attack the periodontal tissue and trigger innate and adaptive immune responses in the host (36, 37). Researches show that periodontitis occurs when the inflammatory process continues, resulting in the formation of periodontal pockets and the loss of alveolar bone. Neutrophil migration into the gingival crevicular by Chemokines such as interleukin-8 induce neutrophils to migrate to the gingival crevicular fluid and kill pathogens, which is a process of innate immunity (38, 39). The loss of neutrophils leads to a loss of innate immunity, which perpetuates the presence of microorganisms in the gingival sulci and changes the composition of the oral microbiome (40, 41). This concomitant dysbiosis leads to the homeostasis imbalance between the host and the microbe, and the microbe loses immune control, resulting in early-onset periodontitis (41). Therefore, patients with neutrophil-associated primary immunodeficiencies can present with severe periodontitis (42). Patients with neutropenia and neutrophil defects inevitably develop early-onset periodontitis (41).

In addition, neutropenia can also cause immune dysregulations. Neutrophils have been historically associated with antimicrobial functions in acute infections but are now appreciated as functionally versatile cells with critical roles in chronic inflammation. Neutrophils are necessary for important immunomodulatory functions, and a lack of neutrophils in periodontium leads to dysregulated excessive production of interleukin-17, which drives persistent inflammatory so that continuous inflammatory bone loss (43).

In this case study, neutrophil adhesion defects were not evaluated because clinically the patient had no skin abnormalities as described in some of these disorders. The patient had a quantitative defect of white blood cells. A qualitative abnormality was not investigated, as it is not usually done when neutrophil counts are low (because neutropenia is sufficient to put one at risk for infection).

The HPS-2 subtype is distinguished by neutropenia (12), which might explain why the patient in this case manifested severe periodontitis at age 13.

Patients with neutropenia and immune deficiency had better require periodic periodontal examinations, which are feedback on systemic immune function (44). An HPS patient can receive non-surgical periodontal treatment (NSPT) and regular maintenance. But neutrophilic deficiency leads to poor efficacy of conventional periodontal treatment (45). Studies have shown that patients with neutropenia have a positive response to human granulocyte colony-stimulating factor (G-CSF) (5, 11, 44, 46, 47). The literature has also suggested that HPS-2 patients need hematologic follow-up to identify when G-CSF therapy is needed (5). When the blood neutrophil count is restored to normal with G-CSF, the patient's ability to anti-infection will be greatly improved (44, 46). Therefore, the use of G-CSF can improve the efficacy of periodontal treatment for HPS-2 patients. Dental fear should also be considered when treating dental problems in patients with HPS-2, especially in children and adolescents (48). Studies have shown that ‘drilling with handpiece' and ‘injecting the anesthetic' were the most important factors contributing to dental fear (49). A high level of dental fear may lead to poor therapeutic efficacy. Therefore, the non-injectable anesthetic gel could be considered during NSPT (20). In addition, HPS-2 patients need to take regular dental examinations and maintain good oral hygiene by using an extra-soft toothbrush and conservative brushing technique (44, 45, 50). Glasses with 99 UV filters are also recommended for HPS-2 patients during dental treatment to protect their eyes from unpleasant light stimulus (50).

Studies have confirmed that periodontal disease is related to systemic diseases, and periodontal disease is a manifestation of some systemic diseases (51–53). This study illustrates the importance of diagnosing periodontal disease as a possible indicator of underlying systemic disease. Screening for systemic disease is needed when patients develop unusual, generalized periodontal disease, as the oral disease may be the first or only manifestation of systemic abnormalities. Therefore, as a dentist, especially a periodontist, it is necessary to have a holistic view when treating periodontal diseases.

So far, only one piece of literature reported a 21-year-old male diagnosed with HPS-2 suffered from aggressive periodontitis. But this patient wore orthodontic brackets, and this study did not rule out possible damage to periodontal tissue caused by orthodontic factors (15). This case study is the first to describe a case of HPS-2 with severe periodontitis in an adolescent and described the periodontal status in detail. Although some achievements have been made, there are still some limitations. The relationship and internal mechanism between periodontitis and neutropenia caused by *AP3B1* mutation need to be further explored, which may shed light on the function and underlying mechanism of neutrophils in periodontitis.

## Data availability statement

The authors declare that the materials described in the article, including all relevant raw data, will be freely available to any scientist wishing to use them for non-commercial purposes, without breaching participant confidentiality. All data generated or analyzed during this study are included in this published article.

## Ethics statement

The studies involving human participants were reviewed and approved by Central South University. Written informed consent to participate in this study was provided by the participants' legal guardian/next of kin. Written informed consent was obtained from the minor(s)' legal guardian/next of kin for the publication of any potentially identifiable images or data included in this article. Patients give informed consent to the use of their blood samples and clinical data for scientific research and publication.

## Author contributions

JC and YY designed the research with WL. JC and YY collect and analyze the data and wrote the manuscript. All authors revised the manuscript and approved the manuscript.

## Funding

This work was supported by the Natural Science Foundation of Hunan Province for Young Scientists (2020JJ5404), the Hunan Health Commission Research Grant (2020080, 202108011054), the Young Teacher's Institutional Grant from Xiangya School of Stomatology and Xiangya Stomatological Hospital, Central South University (2018YQ02 and 2019YQ01), the Open Sharing Fund for the Large scale Instruments and Equipment of Central South University (CSUZC202107), and the College Students' Innovative Entrepreneurial Training Plan Program (XCX2021044), China.

## Conflict of interest

The authors declare that the research was conducted in the absence of any commercial or financial relationships that could be construed as a potential conflict of interest.

## Publisher's note

All claims expressed in this article are solely those of the authors and do not necessarily represent those of their affiliated organizations, or those of the publisher, the editors and the reviewers. Any product that may be evaluated in this article, or claim that may be made by its manufacturer, is not guaranteed or endorsed by the publisher.
